# “Masculine” Describes Gender Expressions, Not Neurobiologies: Response to Dutton and Madison (2020)

**DOI:** 10.1007/s13178-020-00502-5

**Published:** 2020-09-28

**Authors:** Reubs J Walsh

**Affiliations:** Department of Clinical, Neuro- and Developmental Psychology, Vrije Universiteit Amsterdam, Amsterdam, The Netherlands

**Keywords:** Gender, Identity, Transgender, Autism, Neuroimaging, Androgens, Sex, Masculinization

## Abstract

This letter is a response to “Gender Dysphoria and Transgender Identity Is Associated with Physiological and Psychological Masculinization: a Theoretical Integration of Findings, Supported by Systematic Reviews” by Dutton and Madison (2020), which relies on theorisations for which substantial counter-evidence exists, fails to engage with these or other criticisms of the theories upon which it seeks to build, and reaches conclusions that contradict existing evidence. Furthermore, the original theorisations contained in Dutton and Madison (2020), and the conclusions drawn from those theorisations, risk causing serious harm to already-marginalised groups.

Dear Editor,

The recent publication of “Gender Dysphoria and Transgender Identity Is Associated with Physiological and Psychological Masculinization: a Theoretical Integration of Findings, Supported by Systematic Reviews” (Dutton & Madison, 2020) casts doubt on the ability of current standards of peer-review to weed out low-quality research that harms marginalised people. In their conclusion, the authors correctly note that their theorisation of transgender identities “suggests that irreversible treatments should be very restrictively employed, as they may not resolve the underlying cause of the experienced problems, and may, instead, lead to further problems for these individuals.” This is a falsifiable prediction of their hypothesis, and in fact, has already been falsified by innumerable studies showing that psychiatric outcomes for transgender patients are vastly improved by gender-affirmation surgeries (Wernick, Busa, Matouk, Nicholson, & Janssen, 2019) and social transition (Olson, Durwood, DeMeules, & McLaughlin, 2016). It is reasonable to assume that a significant proportion of the participants in these follow-up studies were autistic, assuming the overlap indeed exists. Furthermore, although there remains a lack of specific follow-up studies with autistic people who have undergone a gender transition, the consensus among expert clinicians, most of whom are cisgender, is that although in some cases, autistic traits may complicate the patient’s decision-making, and additional support is required, autism is not, in and of itself, a contra-indication for social or medical gender transition (Strang et al., 2018).

In addition to this, the idea that the brains of men and women differ for innate biological, rather than sociocultural, reasons, is hard to defend (e.g. Rippon, 2019). Despite significant and sustained efforts to identify consistent neuroanatomical correlates of sex and gender, the kinds of gross-anatomical differences discussed in the paper have not yet been established (e.g. Joel et al., 2015), and indeed, a recent study found that when gold-standard correction for sex-related differences in intracranial volume (i.e. gross anatomy of the skull itself) is used, the apparent magnitude and consistency of gender differences drops dramatically, such that machine learning (ML) classifiers perform barely above chance—at a level that could be explained by imperfections in the intracranial volume correction just as easily as by innate sex-differences (Segura et al., 2020). Relatedly, when individual traits (including both morphological and functional/behavioural measures) are classified as “typically-male” or “typically-female”, the vast majority are an unbiased mixture of “male” and “female” traits (Joel et al., 2015). Furthermore, when an ML classifier is trained on American participants and then tested on Chinese or Israeli participants, this drop in accuracy is once again observed (Joel et al., 2018), suggesting a role for culture in the creation of those differences that do exist. Whilst it is impossible to prove the absence of any effect, this work shows that innate sex-related effects on brain morphology and connectivity are very small if they exist at all.

The target paper barely engages with the substantial literature of evidence against the extreme male brain (EMB) hypothesis of autism aetiology (e.g. Barbeau, Mendrek, & Mottron, 2009; Falter, Plaisted, & Davis, 2008; Voracek & Dressler, 2006), for example, the evidence that 2D:4D ratios do not correlate with autism measures developed by the same group that first proposed EMB (Voracek & Dressler, 2006), and that congenital adrenal hyperplasia (in which foetal testosterone is substantially elevated) does not correspond to an increased incidence of autism (Barbeau et al., 2009). Evidence against the Blanchard typology is also ignored (Moser, 2009, 2010; Serano, 2020; Veale, 2015; Veale, Clarke, & Lomax, 2008), for example the evidence that cisgender women experience “autogynephilic” fantasies similar or identical to those experienced by transgender women (Moser, 2009; Veale et al., 2008). There are also several factual errors, the most significant of which is probably the assertion that people assigned male at birth are overrepresented in the transgender population (Cheung et al., 2018).

Finally, therefore, the suggestion that trans people are “masculinised” based on the overlap with autism is no more valid than another of the authors’ assertions that a 2% difference in the rate of left-handedness between the sexes renders handedness a meaningful correlate of sex. A more parsimonious proposition is that autistic people are more likely to identify as trans due to differences in perception and cognition leading to a reduction in the likelihood that social conditioning will prevent them from becoming aware of their gender identity when it differs from the gender assigned to them at birth (Jackson-Perry, 2020; Walsh, Krabbendam, Dewinter, & Begeer, 2018).

Considering these numerous scientific failures, Dutton and Madison (2020) represent a perhaps-unintentional attack on a vulnerable minority which (ab)uses the power and authority of academic science to provide credibility to a pathologizing and cisgenderist theory with little-to-no scientific or academic merit.
